# Blood pressure management in ischemic stroke patients undergoing mechanical thrombectomy

**DOI:** 10.1186/s42466-023-00238-8

**Published:** 2023-03-30

**Authors:** Michael De Georgia, Theodore Bowen, K. Rose Duncan, Alex Bou Chebl

**Affiliations:** 1 Department of Neurology, Case Western Reserve University School of Medicine, Cleveland, OH USA; 2 Department of Neurology, MetroHealth Medical Center, Cleveland, OH USA; 3 Department of Neurology, Henry Ford Medical Center, Detroit, MI USA

**Keywords:** Ischemic stroke, Blood pressure, Endovascular, Mechanical thrombectomy

## Abstract

The relationship between presenting blood pressure in acute ischemic stroke patients and outcome is complex. Several studies have demonstrated a U-shaped curve with worse outcomes when blood pressure is high or low. The American Heart Association/American Stroke Association guidelines recommend values of blood pressure < 185/110 mmHg in patients treated with intravenous t-PA and “permissive hypertension” up to 220/120 mmHg in those not treated with intravenous t-PA. The optimal blood pressure target is less clear in patients undergoing mechanical thrombectomy. Before thrombectomy, the guidelines recommend a blood pressure < 185/110 mmHg though patients with even lower systolic blood pressures may have better outcomes. During and after thrombectomy, the guidelines recommend a blood pressure < 180/105 mmHg. However, several studies have suggested that during thrombectomy the primary goal should be to prevent significant low blood pressure (e.g., target systolic blood pressure > 140 mmHg or MAP > 70 mmHg). After thrombectomy, the primary goal should be to prevent high blood pressure (e.g., target systolic blood pressure < 160 mmHg or MAP < 90 mmHg). To make more specific recommendations, large, randomized-control studies are needed that address factors such as the baseline blood pressure, timing and degree of revascularization, status of collaterals, and estimated risk of reperfusion injury.

## Introduction

Ischemic stroke is the fifth leading cause of death in the United States, second leading cause of death in the European Union, and leading cause of long-term disability in both [1]. While hypertension is the most important modifiable risk factor [1], the relationship between presenting blood pressure and stroke outcome is complex. Several studies have found a U-shaped relationship, with worse outcomes when presenting blood pressures are high or low [2–4]. However, trials to determine if reducing blood pressure after ischemic stroke is beneficial have yielded mixed results. To complicate matters further, there are limited and conflicting data regarding which measurement, systolic blood pressure (SBP), diastolic blood pressure (DBP), or mean arterial pressure (MAP), is the best predictor of outcome or even which kind of analysis, mean, maximum and minimum, or the difference between maximum and minimum. Independent of the blood pressure level, higher blood pressure variability (BPV) after ischemic stroke, that is, fluctuations over time, either beat-to-beat, minute-to-minute, or hour-to-hour, is also associated with poor functional outcome and death [5].

In 2015, five randomized controlled trials established the safety and efficacy of endovascular mechanical thrombectomy for acute ischemic stroke from large vessel occlusion [6–10]. Multiple factors influence the effect of blood pressure on outcomes in these patients, including the baseline blood pressure, size of ischemic penumbra, degree of collateral flow, clot burden, status of recanalization, timing of blood pressure measurement and intervention, and risk of reperfusion injury and hemorrhagic transformation. Given all this complexity, the optimal blood pressure management in this patient population is uncertain. The following is a review of blood pressure and acute ischemic stroke to a provide a general approach to blood pressure management in patients undergoing mechanical thrombectomy.

## Blood pressure measurement

Blood pressure is the driving force of blood flow through the tissue bed and is generated in the aorta from the contraction of the left ventricle. SBP is the maximal aortic pressure following ejection of blood from the ventricle, whereas DBP is the lowest aortic pressure just before the ventricle ejects blood again. MAP is the average pressure generated during one ventricular contraction. Using invasive intraarterial blood pressure monitoring, the MAP is calculated by integrating the area under the curve of the pressure-time waveform. With noninvasive, oscillometric blood pressure monitoring, the MAP is calculated most commonly by the equation DBP + 0.333(SBP-DBP) [11].

Invasive arterial blood pressure monitoring represents the gold standard as it provides continuous measurement and is preferred for critically ill patients. Noninvasive oscillometric monitoring is more common in acute ischemic stroke patients but is intermittent rather than continuous. In addition, oscillometric SBP measurements tend to be *lower* than invasive monitoring at high blood pressures and *higher* than invasive monitoring at low blood pressures. In contrast, MAPs are similar when assessed by oscillometry and invasive techniques over a range of pressures [12].

## Blood pressure after ischemic stroke

High blood pressure is common in patients with acute ischemic stroke, with over half having an SBP > 160 mmHg [13]. Blood pressure usually decreases spontaneously over the first seven days [14]. The reasons for high blood pressure are complex and thought to stem partly from the “cerebral ischemic response,” a derivative of the diving reflex in mammals. Specifically, cerebral ischemia triggers an autonomic response in the rostral ventrolateral medullary nucleus. When stimulated, these neurons project to the spinal cord sympathetic preganglionic fibers to increase blood pressure and through the subthalamic nucleus to the cortex causing local vasodilatation to increase cerebral blood flow [15]. Low blood pressure, on the other hand, is uncommon in acute ischemic stroke. Possible reasons include hypovolemia, reduced cardiac output from heart failure, myocardial ischemia, arrhythmias, aortic dissection, sepsis, and hypothermia [16, 17].

## Blood pressure and outcome

### The U-shaped curve

**Fig. 1:**
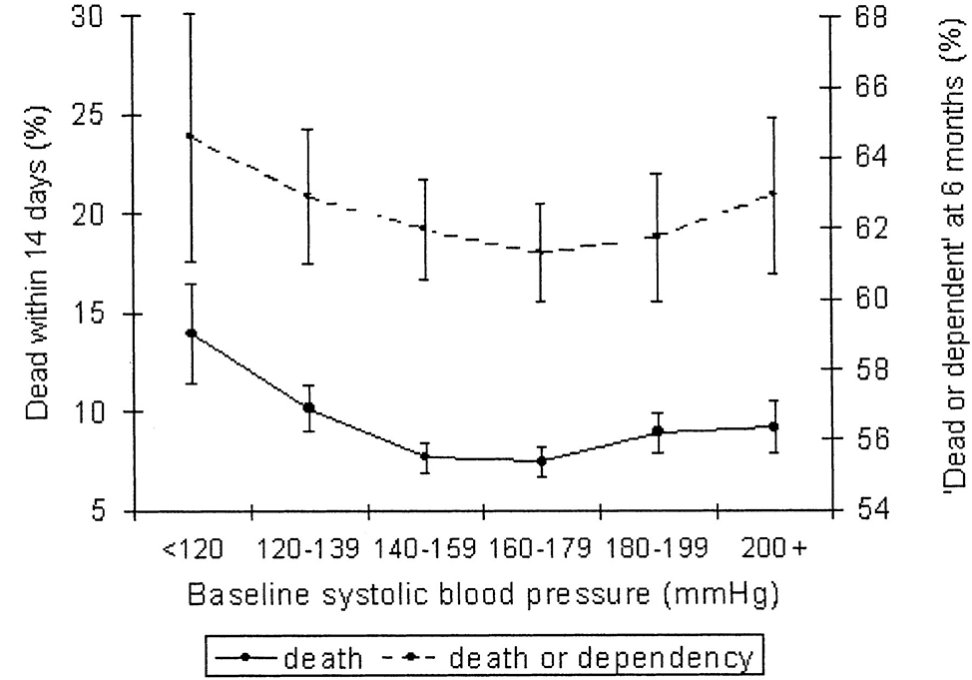
Proportion of patients from the International Stroke Trial who died within 14 days (solid lines) or were dead or dependent at six months (dashed lines) by baseline SBP. Circles and squares indicate the mean percentage of patients who had died within 14 days and patients who had died or were dependent at six months, respectively, within each blood pressure subgroup; 95% CIs are represented by T bars. (From Leonardi-Bee J, Bath PMW, Phillips SJ, et al. Stroke. 2002;33:1315–1320 with permission from publisher, https://www.ahajournals.org/doi/10.1161/01.STR.0000014509.11540.66).

Several studies have demonstrated a U-shaped relationship between presenting blood pressure and stroke outcome. For example, in the International Stroke Trial, both patients with SBPs < 120 mmHg and > 200 mmHg had worse outcomes. There appeared to be a “sweet spot” of optimal SBP around 150 mmHg, where the risk of death within 14 days or death or dependency at six months was the lowest. For example, for each 10 mmHg below SBP 150 mmHg, there was a 17.9% increase in early death, and for each 10 mmHg above SBP 150 mmHg, a 3.8% increase in early death [2] (Fig. 1). Other studies have confirmed this relationship. Vemmos and colleagues, in a prospective observational study of 1,121 patients, identified 130 mmHg as the optimal SBP [3] while Castillo and colleagues found a higher optimal SBP of 180 mmHg [4]. Cerebral autoregulation and recanalization status are two mechanisms underlying this U-shaped curve [18].

### Cerebral autoregulation status

Normal cerebral autoregulation occurs when small arterioles dilate and constrict depending on the cerebral perfusion pressure (CPP) (the difference between MAP and intracranial pressure). As a result, cerebral blood flow stays constant over a wide range of CPPs. When the CPP is very low, the arterioles dilate to their maximal diameter, and when very high, they constrict to their minimal diameter. During these ranges, the relationship between CPP and CBF becomes linear. Autoregulation is a complex physiological process that includes myogenic, autonomic, and metabolic mechanisms. In ischemic stroke, direct damage to the myosin within the vessel wall can impair this process [19]. Brainstem lesions can also impair cerebral autoregulation [20]. Patients may, therefore, be at risk for *hypoperfusion* with hypotension. In addition, because chronic hypertension, common among stroke patients, results in a rightward shift in the autoregulation curve, patients may be at risk for hypoperfusion even with modest drops in blood pressure. Patients may also be at risk for *hyperperfusion* with hypertension, which can lead to endothelial damage, cerebral edema, and intracranial hemorrhage, especially after the administration of intravenous tissue plasminogen activator (t-PA) or cerebral revascularization [21]. Bedside tools to assess cerebral autoregulation include transcranial doppler ultrasound and near-infrared spectroscopy [22].

### Recanalization status

**Fig. 2:**
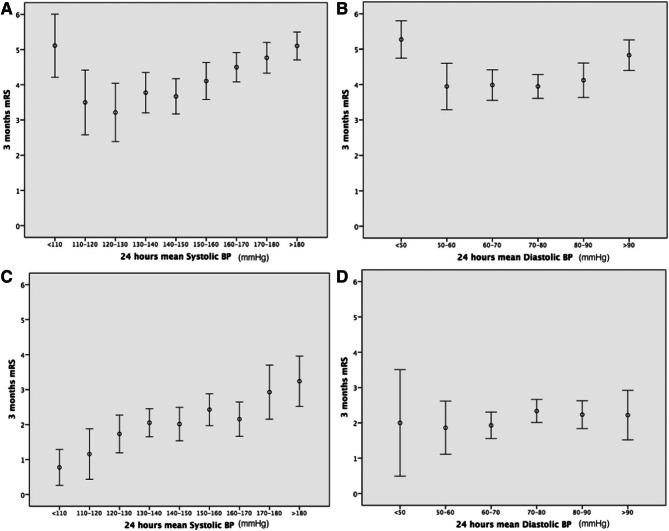
Relationship between blood pressure (BP) in the first 24 h post ischemic stroke and functional outcome at 3 months (through modified Rankin Scale [mRS]). **A** and **B**, Nonrecanalyzed patients. **C** and **D**, Recanalyzed patients. Error bars represent the 95% confidence interval for mean. (From Martins AI, Sargento-Freitas J, Silva F, et al. Stroke. 2016;47:1571–1576 with permission from publisher, https://www.ahajournals.org/doi/full/10.1161/STROKEAHA.115.012544)

In addition to cerebral autoregulation, recanalization status also modulates the effect of blood pressure on. In a study of 674 patients treated with intravenous t-PA or endovascular intervention, Martins and colleagues showed a U-shaped relationship between blood pressures in the first 24 h and 3-month functional outcomes in *non-recanalyzed* patients but a linear relationship in *recanalyzed* patients, with higher blood pressures correlating with worse outcomes [23] (Fig. 2). Reasons that high blood pressure are associated with a worse outcome may differ between these two groups. In *non-recanalyzed* patients, hypertension may simply reflect a persistently occluded vessel and a more severe stroke [24]. In angiographic-based studies, recanalization is synchronous with a drop in blood pressure [25, 26]. Thus, high blood pressure in acute ischemic stroke patients may be the body’s natural response to an occluded vessel. In contrast, in *recanalyzed* patients, hypertension may lead to a worse outcome because of cerebral edema, reperfusion injury and hemorrhagic transformation.

## Blood pressure management after ischemic stroke

### Low blood pressure

Treating frank hypotension in acute ischemic stroke patients is indicated, and guidelines recommend that correctable causes (e.g., hypovolemia, cardiac arrhythmias, hypothermia) be sought and reversed [27]. However, whether *inducing hypertension* with volume expansion or vasopressors in normotensive patients in an attempt to augment cerebral blood flow is beneficial is uncertain. Some animal models have demonstrated reduced infarct volumes with induced hypertension [28], but only a handful of small clinical studies have shown improved outcomes [29, 30]. Most have concluded that inducing hypertension is relatively safe [31]. Theoretically, increasing blood pressure may augment cerebral blood flow to the ischemic penumbra and mitigate injury. However, patients with little or no penumbra may not benefit from blood pressure elevation. Few studies have assessed the benefit of induced hypertension in patients without an ischemic penumbra [31]. Also, other than one study (Hillis and colleagues [30]), the eloquence of the ischemic tissue has not been considered. As the eloquence of the brain region affected by the stroke significantly impacts the outcome, not considering this could limit the power of any study of induced hypertension to find a benefit. Regarding induced hypertension, the American Heart Association/American Stroke Association (AHA/ASA) guidelines urge caution writing, “the usefulness of drug induced hypertension in patients with acute ischemic stroke is not well-established… Induced hypertension should be performed in the setting of clinical trials” [32].

### High blood pressure

#### Patients treated with intravenous t-PA

Elevated blood pressure in the immediate post-stroke period is far more common. Before intravenous t-PA can be administered, the AHA/ASA guidelines require a blood pressure of < 185/110 mmHg and maintenance of blood pressure < 180/105 mmHg for the first 24 h following thrombolysis [27]. Whether more intensively lowering blood pressure improves outcome isn’t clear. The Enhanced Control of Hypertension and Thrombolysis Stroke Study (ENCHANTED), which randomized patients to an intensive blood pressure target (SBP 130–140 mmHg) vs. the guideline-recommended target (< 180 mmHg), did not show an improved outcome despite a lower incidence of intracranial hemorrhage. This may be because the blood pressure difference between the two groups was small (only 5 mmHg) [33]. These findings were unchanged when assessed within lacunar and non-lacunar stroke subgroups [34].

#### Patients not treated with intravenous t-PA

For patients not treated with intravenous t-PA, the optimal blood pressure target has yet to be well established. Multiple studies have shown that hypertension during the acute phase of stroke is associated with worse outcomes [21, 35, 36], but whether reducing high blood pressure improves outcome isn’t certain. On the contrary, reducing high blood pressure may lead to neurological deterioration [37]. In the study by Castillo and colleagues, it was a drop in SBP of > 20 mmHg that most impacted neurological deterioration and correlated with increased infarct volumes [4]. Fearing early deterioration with active blood pressure lowering more than the elevated blood pressure itself, the AHA/ASA guidelines recommend “permissive hypertension,” unless the blood pressure is “markedly elevated” (i.e., > 220/120 mmHg), at which point it’s recommended to “lower the blood pressure by 15%” [32].

Unfortunately, the results of randomized controlled trials have been mixed and have only led to confusion. For example, the Scandinavian Candesartan Acute Stroke Trial (SCAST) compared candesartan to placebo in hypertensive patients (SBP > 140 mmHg) and found that active blood pressure lowering did lead to a trend towards a worse outcome [38]. However, in the Controlling Hypertension and Hypotension Immediately Post Stroke (CHHIPS) trial, in which hypertensive patients (SBP > 160 mmHg) were randomized to actively blood pressure lowering or placebo, not only was there no early deterioration observed in those with active blood pressure lowering but a nearly 50% reduction in mortality at three months [39]. The results of the Continue or Stop Post-Stroke Antihypertensives Collaborative Study [40], in which hypertensive patients were randomized to either continuation or cessation of their home antihypertensive medications, showed no difference in the rate of death or dependency at two weeks [40]. A 2015 meta-analysis of 13 randomized controlled trials and 12,703 patients found that blood pressure lowering within three days of stroke neither improved nor worsened outcomes [41].

Whether lowering elevated blood pressure after acute stroke helps or hurts may depend on both the degree and timing of the pressure reduction. Not surprisingly, significant blood pressure reduction is more detrimental than more modest blood pressure reduction. For example, a secondary analysis of SCAST showed that a large systolic decrease of > 28 mmHg was primarily linked to the poor outcome [42]. Similarly, the Intravenous Nimodipine West European Stroke (INWEST) trial, comparing nimodipine with placebo, was halted early due to higher rates of unfavorable neurologic outcomes in the treatment arm, but this was attributed to very low DBPs [43]. In another trial, high-dose (but not low-dose) intravenous nimodipine was associated with an increased rate of death and dependency [44].

Regarding timing, somewhat counter-intuitively, in SCAST, the later treatment was started, the more candesartan harmed patients, whereas those treated within 6 h seemed to benefit [38]. Also, in the INWEST trial, treatment with nimodipine was associated with a worse outcome when given 24 h after stroke onset, had no effect when given 12 to 24 h, but had a better outcome when given within 12 h. Unfortunately, except in patients receiving intravenous t-PA, it’s not entirely clear what to do with blood pressure elevations in ischemic stroke.

## Blood pressure management in patients undergoing mechanical thrombectomy

Mechanical thrombectomy adds another variable to an already complex picture. A key factor in the effect of blood pressure on outcome in patients undergoing mechanical thrombectomy is the timing of blood pressure measurement, that is pre-, intra-, and post-procedural.

### Pre-procedural blood pressure

**Fig. 3:**
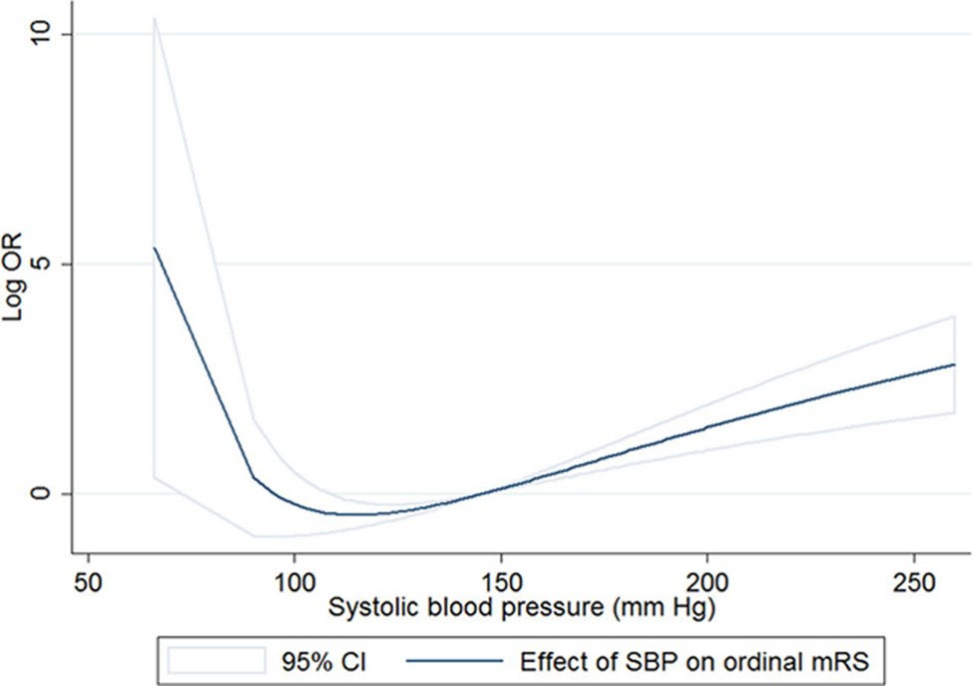
Relation of SBP with functional outcome (modified Rankin Scale [mRS] score after 90 days) in the total Multicenter Randomized Clinical Trial of Endovascular Treatment of Acute Ischemic Stroke in the Netherlands (MR CLEAN) population. CI indicates confidence interval. (From Mulder MJHL, Ergezen S, Lingsma HF, et al. Stroke. 2017;48:1869–1876 with permission from publisher, https://www.ahajournals.org/doi/full/10.1161/STROKEAHA.116.016225)

All the endovascular trials included patients eligible for intravenous t-PA before the intervention. Therefore, the pre-procedural blood pressure target followed the AHA/ASA guidelines of blood pressure of < 180/105 mmHg [27]. A post-hoc analysis of MR CLEAN demonstrated a familiar U-shaped curve, with low and high baseline SBPs associated with poor functional outcomes. While mechanical thrombectomy was beneficial regardless of the pre-procedural blood pressure, the best outcomes occurred in those with a mean SBP of 120 mmHg (Fig. 3). Higher SBP was also associated with a lower probability of successful reperfusion and a greater risk of symptomatic hemorrhagic transformation [45]. In a retrospective analysis of 378 patients, Cho and colleagues also showed that patients with good outcomes had lower baseline SBPs (133.6 mmHg vs. 139.0 mmHg) [46]. Goyal and colleagues also demonstrated a similar trend toward better 90-day functional outcomes in those with lower baseline systolic blood pressures (151 mmHg vs. 165 mmHg) [47].

### Intra-procedural blood pressure

Blood pressure management during mechanical thrombectomy is the least studied time frame. In most of the endovascular trials, intra-procedural blood pressure management protocols were absent. In the EXTEND-IA trial [9], the protocol recommended that investigators maintain a “stable” blood pressure. The most detailed protocol was in the ESCAPE trial [10], in which investigators were to maintain the SBP ≥ 150 mmHg while the blood vessel was occluded to promote collateral flow. No conclusions can be drawn from these trials regarding optimal blood pressure management.

### Low blood pressure

Several studies have indirectly examined the role of intra-procedural blood pressure by comparing outcomes following general anesthesia vs. conscious sedation, focusing mainly on low blood pressure. Patients receiving general anesthesia had lower blood pressures and worse outcomes [48–50], though they were also older, had more comorbidities, more severe strokes, and lower rates of recanalization. Thus, the causative effect of low blood pressure on outcome was unclear.

There have been three randomized trials comparing general anesthesia with conscious sedation: General or Local Anesthesia in Intra Arterial Therapy (GOLIATH) trial [51], Sedation vs. Intubation for Endovascular Stroke Treatment (SIESTA) trial [52], and the Anesthesia During Stroke (AnStroke) trial [53]. Analysis of individual blood pressure and outcome data from these trials found that regardless of the type of anesthesia administered, intra-procedure MAP < 70 mmHg for more than 10 min was associated with a higher 90-day modified Rankin Scale score [54]. Post-hoc analyses of the MR CLEAN trial also demonstrated a dose-dependent association between blood pressure reductions and unfavorable outcome among patients undergoing general anesthesia [55] as well as conscious sedation [56].

Several other studies confirmed that, after controlling for comorbidities and regardless of the type of anesthesia, *substantial* intra-procedural blood pressure drops (e.g., falls in MAP > 40% from baseline) were associated with worse neurologic outcomes [57] and increased infarct growth [58]. Contributing to the association of intra-procedural low blood pressure with worse outcome may also be disturbed blood flow through the catheter and the vasoactive effect of iodinated contrast agent [59]. In addition, low blood pressure leads to low shear stress and turbulent blood flow [60].

It’s likely that the degree of collateralization contributes to the lower blood pressure threshold that can be tolerated. Patients with poor collaterals may be at especially high risk of hypoperfusion such that even small blood pressure drops (e.g., > 10% drop from baseline) can increase the chances of a poor outcome [49]. A post-hoc analysis of the Contact Aspiration vs. Stent Retriever for Successful Revascularization (ASTER) trial showed intra-procedural hypotension was associated with worse functional outcomes among patients with no posterior communicating artery [61]. Patients with better collateral circulation tolerate greater drops in blood pressure [62].

### High blood pressure

While hypotension (absolute or relative) may be associated with a worse outcome, the association between intra-procedural hypertension and outcome is more complicated. In addition to MAP < 70 mmHg, analysis of the GOLIATH, SIESTA, and AnStroke trials also found that MAP > 90 mmHg for more than 45 min was also associated with worse outcomes. Similarly. John and colleagues retrospectively reviewed 147 patients who underwent endovascular intervention and found that a mean maximum intraprocedural SBP of 180.9 mmHg was an independent predictor of poor outcome [63]. Similarly, Chen and colleagues reported worse outcomes with higher intraprocedural blood pressure values. Specifically, pre-recanalization thresholds of SBP > 163 mmHg and MAP > 117 mmHg predicted poor functional outcome [64].

Some have suggested that hypertension itself *leads* to vessel occlusion and contributes to a poor recanalization rate. In one study, patients presenting with SBP > 185 mmHg were more likely to show persistent vessel occlusion or partial recanalization compared with those with lower blood pressures [65]. In a multivariate analysis of predictors of successful recanalization using the Mechanical Embolus Removal in Cerebral Ischemia (MERCI) device, Nogueira and colleagues also showed that SBP > 150 mmHg was associated with a lower likelihood of recanalization [66]. It’s been hypothesized that resistance to recanalization may be the result of a reduction in the endogenous capacity for fibrinolysis along with increased Plasminogen Activator Inhibitor, fibrinogen, serum viscosity, and von Willebrand Factor [67].

**Fig. 4:**
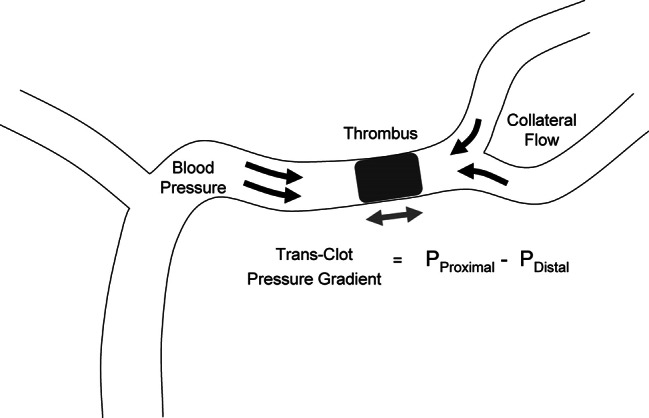
Potential hydromechanical forces influencing the degree of thrombus impaction. P_Proximal_ pressure on the proximal aspect of the clot; P_Distal_ pressure on the distal aspect of the clot. P_Proximal_ pressure is increased by higher arterial pressure. P_Distal_ pressure is increased by higher retrograde arterial pressure (e.g., collateral flow). (From Nogueira RG, Liebeskind DS, Sung G, et al. Stroke 2009;40:3777–3783 with permission from the publisher, https://www.ahajournals.org/doi/10.1161/strokeaha.109.561431)

The degree of collateral flow may also interact with blood pressure and affect hydromechanical forces on the clot. Specifically, in patients with poor collateral circulation, there is reduced pressure distal to the clot. Because the pressure proximal to the clot is relatively higher, clot compaction might be greater, making thrombectomy more difficult. As several studies have shown an association between higher admission blood pressure and poorer collaterals, it suggests the hypertensive response may be needed to support perfusion to the ischemic tissue even at the expense of increasing clot compaction [66]. Conversely, in those with good collateral circulation, the pressure distal to the clot is higher, counterbalancing the proximal pressure, leading to less clot compaction [68] (Fig. 4). In theory, patients with good collaterals may benefit from blood pressure reduction, which could facilitate clot retrieval.

Other studies have shown mixed results regarding whether lower or higher blood pressure during mechanical thrombectomy is better. For example, Seby and colleagues showed a lower maximum SBP (164.6 mmHg vs. 180.9 mmHg) was independently associated with a better outcome [63]. Conversely, Pikija and colleagues found that patients with more frequent SBP excursions > 120% above their baseline had better functional outcomes and lower infarct volumes [69].

Putting all of this together, given the pathophysiological complexity and conflicting data, it’s difficult to make sweeping generalizations. But intra-procedural *hypotension* appears to be linked to a worse outcome. Even small drops in blood pressure (depending on the status of autoregulation, recanalization, and collaterals) may be associated with worse outcomes. Thus, preventing excessive drops in blood pressure during mechanical thrombectomy (e.g., targeting a SBP > 140 mmHg) should be a primary goal. Intraprocedural hypertension also seems to linked with a worse outcome. A sweet spot of SBP 140–160 mmHg may be the optimal target but prospective trials are needed to confirm this.

### Post-procedural blood pressure

Post-procedural blood pressure targets have also varied between studies. In the ESCAPE trial, “normotension” after reperfusion was targeted [10] and REVASCAT, a blood pressure of < 160/90 mmHg was targeted for patients with a TICI 2b flow or greater [8].

Numerous observational studies have shown that higher post-procedural blood pressures generally correlate with worse functional outcomes, especially in patients with poor collaterals [70, 71]. Whether reducing blood pressure improves outcome is unknown. One large retrospective study by Anadani and colleagues found that reduction in SBP within the first 24 h following successful reperfusion among patients with anterior circulation large vessel occlusion was associated with lower odds of worse outcomes among patients with complete reperfusion, but not those with incomplete reperfusion [72]. In a large retrospective study of 703 patients undergoing mechanical thrombectomy, Cernik and colleagues reported that patients with good outcome had a significantly lower median SBP (131 mmHg vs. 140 mmHg) and maximal SBP (160 mmHg vs. 170 mmHg) [73]. Similarly, the Blood Pressure after Endovascular Therapy for Ischemic Stroke trial (BEST), a prospective, multi-center cohort study that included 446 patients post mechanical thrombectomy, found that a maximum SBP > 158 mmHg was associated with a worse outcome [74].

The relationship between higher post-procedure SBP and poor outcomes is mainly because of greater reperfusion injury, however, high post-procedural blood pressure has been inconsistently associated with hemorrhagic transformation. This is likely because of differences in inclusion criteria (anterior circulation only or both anterior and posterior circulation), recanalization status, and definition of hemorrhagic transformation. For example, Mistry and colleagues, in a study of anterior circulation vessel occlusions, showed that maximum SBP within the first 24 h after thrombectomy independently correlated with a worse outcome at three months and hemorrhagic transformation (symptomatic and asymptomatic) at 48 h. Supporting the theory that hemorrhage is tied to reperfusion injury, hemorrhages occurred at lower mean SBPs in patients who had successful recanalization [75]. A post-hoc analysis of eight MR CLEAN registry sites showed an association between higher maximum SBP in the first six hours post-procedure and worse outcomes [76]. Goyal and colleagues reported on patients with both anterior and posterior circulation vessel occlusions and showed that maximum SBP within 24 h after thrombectomy also independently correlated with worse outcomes but not with hemorrhagic transformation. Only *symptomatic* hemorrhagic transformation was included and successful recanalization occurred in a lower percentage of patients (67% vs. 80%) [77]. Similarly, Anadani and colleagues, in a study of patients with both anterior and posterior circulation vessel occlusions, showed that higher average SBP (> 120 mmHg) within 24 h after mechanical thrombectomy independently correlated with worse outcome but also not hemorrhagic transformation (however, they used an even narrower definition - parenchymal hematoma Type 2) [78]. In the BP-TARGET trial, in which post-thrombectomy patients were randomized to an intensive SBP target (100–129 mmHg) or a standard SBP target (130–185 mmHg), there was no significant reduction in hemorrhagic transformation rates (symptomatic or parenchymal hematoma Type 2) at 24–36 h [79].

**Fig. 5:**
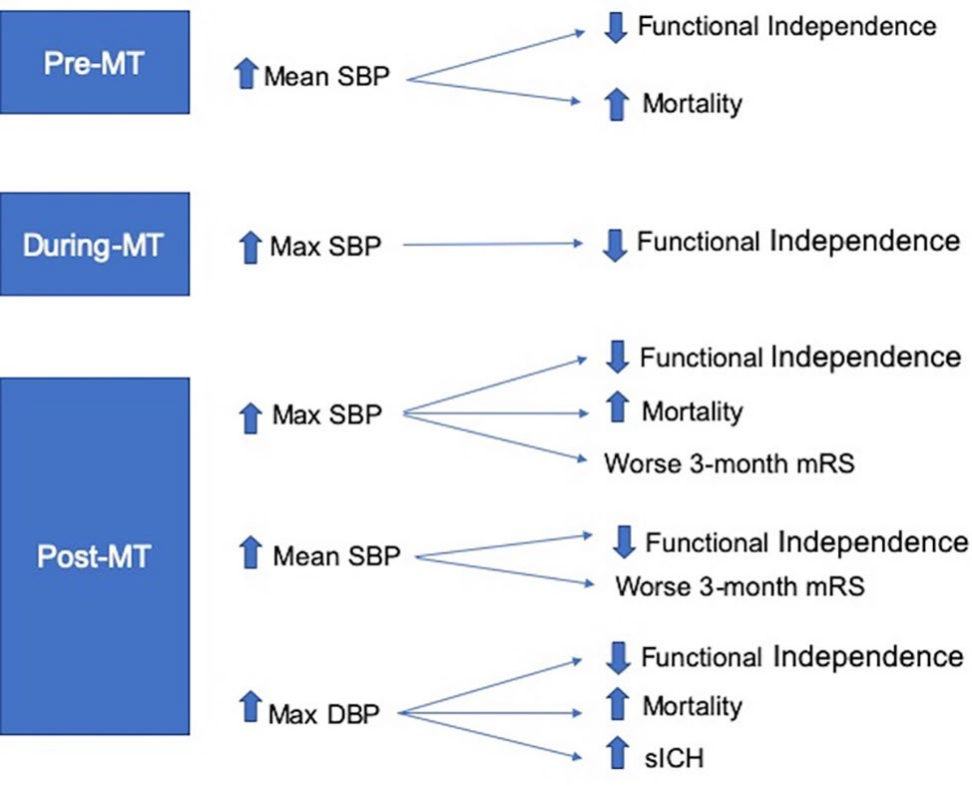
Association of blood pressure and outcome before, during, and after mechanical thrombectomy. (From Malhotra K, Goyal N, Katsano AH, et al. Hypertension 2020;75:730–739 with permission from the publisher, https://www.ahajournals.org/doi/full/10.1161/HYPERTENSIONAHA.119.14230).

A recent meta-analysis of 25 studies and 6,474 patients by Malhotra and colleagues examining pre-, intra-, and post-procedural blood pressure and outcome concluded broadly that, before thrombectomy, higher mean SBP levels were associated with a lower odds of 3-month independence and increased mortality. During thrombectomy, higher maximum SBP levels were associated with lower odds of 3-month independence. There was no association between low SBP and outcome although only three trials [49, 50, 63] were included in this analysis. After thrombectomy, both higher mean SBP levels and maximum SBP levels were associated with lower odds of 3-month independence and increased mortality. Higher maximum DBP levels were also associated with hemorrhagic transformation [80]. (Fig. 5). Current AHA/ASA guidelines recommend a fixed blood pressure target of ≤ 180/105 mmHg during and for 24 h after the procedure (IIa recommendation). [27]

Finally, in a sub study of the BEST trial, higher BPV in the first 24 h after thrombectomy was associated with a worse 90-day outcome, especially for systolic BPV and in patients with successful recanalization. Interestingly, in patients treated with intravenous antihypertensive medications, while the mean blood pressure dropped, the BPV increased [81]. Further research is needed to determine the best method to analyze blood pressure (mean, maximum or minimum, or BPV) and which strategies are best for treatment. For example, calcium channel blockers tend to reduce BPV more than beta-blockers or angiotensin-converting enzyme inhibitors.

## Conclusions

Despite the fundamental relationship between blood pressure and cerebral blood flow, the optimal management of blood pressure in acute ischemic stroke is not straight forward. For patients receiving intravenous thrombolysis, the AHA/ASA guidelines recommend a blood pressure < 185/100 mmHg in order to decrease the risk of hemorrhagic transformation. For those not receiving thrombolysis, the guidelines recommend “permissive hypertension” up to 220/120 mmHg though studies of blood pressure lowering have yielded mixed results. This is likely because of heterogenous patient populations and various other confounding factors including, baseline blood pressure, timing of measurement and intervention, cerebral autoregulation and recanalization status. For ischemic stroke patients undergoing mechanical thrombectomy, the optimal blood pressure target which both maintains cerebral perfusion of ischemic tissue while avoids reperfusion injury and hemorrhagic transformation remains unclear. Some general recommendations are as follows:

- Before mechanical thrombectomy, for patients having received intravenous t-PA, a blood pressure target of < 185 mmHg is recommended, as per AHA/ASA guidelines. Patients with even lower blood pressures (e.g. < 160 mmHg) may have better outcomes (and likely less symptomatic intracranial hemorrhage).
- During mechanical thrombectomy, the primary goal should be to prevent significant *low blood pressure* (e.g., target SBP > 140 mmHg or MAP > 70 mmHg). The upper range target is not clear but patients with very high SBP appear to have a worse outcome and so targeting a SBP in the 140–160 mmHg range may be optimal especially in the setting of good collaterals.
- Following mechanical thrombectomy, the primary goal should be to prevent *high blood pressure* (e.g., target SBP < 160 mmHg or MAP < 90 mmHg) as there is convincing data that higher SBPs lead to a worse functional outcomes and higher risk of hemorrhagic transformation. This is particularly true following successful vessel recanalization. In these patients, lowering the post-procedure blood pressure more aggressively (e.g., SBP < 140 mmHg) may be beneficial.

Ultimately, hemodynamic goals in patients with ischemic stroke undergoing mechanical thrombectomy should be individualized based on the baseline blood pressure, timing and degree of revascularization, status of collaterals, and estimated risk of reperfusion injury. In addition, measurement of the cerebral autoregulation status may also be helpful in targeting an optimal blood pressure. Blood pressure variability is an emerging risk factor poor outcome after ischemic stroke and following mechanical thrombectomy. In the future, large, high-quality randomized-control studies are needed that carefully address these all of these factors.

## Data Availability

Not Applicable.

## Abbreviations

SBP: Systolic Blood Pressure
DBP: Diastolic Blood Pressure
MAP: Mean Arterial Pressure
BPV: Blood Pressure Variability
CPP: Cerebral Perfusion Pressure
t-PA: Tissue Plasminogen Activator
AHA: American Heart Association
ASA: American Stroke Association
ENCHANTED: Enhanced Control of Hypertension and Thrombolysis Stroke Study
SCAST: Scandinavian Candesartan Acute Stroke Trial
CHHIPS: Controlling Hypertension and Hypotension Immediately Post Stroke trial
INWEST: Intravenous Nimodipine West European Stroke Trial
MR CLEAN: Multicenter Randomized Clinical Trial of Endovascular Treatment for Acute Ischemic Stroke in the Netherlands
EXTEND-IA: Extending the Time for Thrombolysis in Emergency Neurological Deficits — Intra-Arterial trial
ESCAPE: Endovascular Treatment for Small Core and Anterior Circulation Proximal Occlusion with Emphasis on Minimizing CT to Recanalization Times trial
GOLIATH: General or Local Anesthesia in Intra Arterial Therapy trial
SIESTA: Sedation vs. Intubation for Endovascular Stroke Treatment trial
ASTER: Contact Aspiration vs Stent Retriever for Successful Revascularization trial
AnStroke: Anesthesia During Stroke trial
MERCI: Mechanical Embolus Removal in Cerebral Ischemia
BEST: Blood Pressure after Endovascular Therapy for Ischemic Stroke trial
